# Clonal expansion of CD4^+^CD8^+^ T cells in an adult patient with *Mycoplasma pneumoniae*-associated Erythema multiforme majus

**DOI:** 10.1186/s13223-021-00520-x

**Published:** 2021-02-10

**Authors:** Sarah M. Volkers, Christian Meisel, Dorothea Terhorst-Molawi, Guido J. Burbach, Dirk Schürmann, Norbert Suttorp, Leif E. Sander

**Affiliations:** 1 Department of Infectious Diseases and Respiratory Medicine, Charité—Universitätsmedizin Berlin, corporate member of Freie Universität Berlin, Humboldt-Universität zu Berlin, and Berlin Institute of Health, Berlin, Germany; 2 Institute of Medical Immunology, Charité—Universitätsmedizin Berlin, corporate member of Freie Universität Berlin, Humboldt-Universität zu Berlin, and Berlin Institute of Health, Berlin, Germany; 3 Department of Immunology, Labor Berlin—Charité Vivantes GmbH, Berlin, Germany; 4 Department of Dermatology, Charité—Universitätsmedizin Berlin, corporate member of Freie Universität Berlin, Humboldt-Universität zu Berlin, and Berlin Institute of Health, Berlin, Germany; 5 Dermatology/Dermato-Oncology Out-Patient Clinic, Vivantes Ambulatory Health Care Centers Berlin-Spandau, Berlin, Germany; 6 German Center for Lung Research (DZL), Berlin, Germany

**Keywords:** Erythema multiforme, *Mycoplasma pneumoniae*-associated rash and mucositis, T cells, CD4^+^ CD8^+^ double-positive T cells, Tissue-resident memory T cells, Mucosal-associated invariant T cells, T effector memory RA^+^ T cells

## Abstract

**Background:**

Erythema multiforme (EM) is an acute, immune-mediated mucocutaneous disease, most often preceded by herpes simplex virus (HSV) infection or reactivation. *Mycoplasma pneumoniae* (Mp) is considered the second major trigger of EM and is often associated with an atypical and more severe presentation of disease, characterized by prominent mucosal involvement. However, contrary to HSV-associated Erythema multiforme (HAEM), immunological mechanisms of Mp-associated EM remain unclear.

**Case presentation:**

We present the case of a 50-year-old male patient presenting with community-acquired pneumonia (CAP) and erythema multiforme majus (EMM). Acute Mp infection was diagnosed by seroconversion, with no evidence of HSV infection as a cause of EMM. We performed immune phenotyping of blister fluid (BF) and peripheral blood (PB) T cells and detected a clonally expanded TCRVβ2^+^ T cell population that was double positive for CD4 and CD8, and expressed the cytotoxic markers granulysin and perforin. This CD4^+^CD8^+^ population comprised up to 50.7% of BF T cells and 24.9% of PB T cells. Two years prior to the onset of disease, the frequency of PB CD4^+^CD8^+^T cells had been within normal range and it gradually returned to baseline levels with the resolution of symptoms, suggesting an involvement of this population in EMM disease pathophysiology.

**Conclusions:**

This report is the first to provide a phenotypic description of lesional T cells in Mp-associated EMM. Characterizing the local immune response might help to address pathophysiological questions and warrants further systematic research.

## Background

Erythema multiforme (EM) is an acute, immune-mediated mucocutaneous disease characterized by typical target or raised atypical target lesions, typically with an acral distribution [1]. EM can occur in patients of all ages, but it is most prevalent in young adults and shows a predominance for the male sex [2, 3]. EM comprises a minor and a major form, with ≤ 1 (Erythema multiforme minus, EMm) or ≥ 2 (Erythema multiforme majus, EMM) mucosal sites involved, respectively [1]. EMM may also be accompanied by general illness such as fever or fatigue [2, 3]. In most cases, EM is preceded by infection/reactivation with herpes simplex virus (HSV) and is thought to be caused by HSV DNA fragments, transported to the skin by Langerhans cell precursors [4, 5]. Expression of certain HSV genes, notably DNA polymerase (pol), by keratinocytes leads to an inflammatory immune response initiated by HSV-antigen specific CD4^+^ T helper cell type 1 cells whose T cell receptor (TCR) repertoire is usually skewed towards usage of the TCRVβ2 chain [5]. EM is self-limited, but may recur in up to 30% of EMm and 10% of EMM patients, respectively [3].

Besides HSV, other pathogens have been associated with EM as well [6], especially *Mycoplasma pneumoniae* (Mp), which is considered the second major cause of EM and the primary cause of EM in children [3]. Mp-associated EM presentation is often atypical and more severe than HSV-associated EM (HAEM), with prominent mucositis and either a non-acral distribution of atypical (larger) targets [3, 7] or only very sparse or even absent cutaneous involvement. The latter condition is referred to as “*Fuchs Syndrome*” or “*Mucosal EMM*” [3]. Mucosal sequelae affecting the ocular or genital region are more frequent in patients with Mp-associated EM than among patients with non-Mp-associated EM [7].

EM needs to be distinguished from Stevens-Johnson syndrome/Toxical Epidermal Necrolysis (SJS/TEN). EM and SJS/TEN were previously viewed as two shades of a shared syndrome, but are now considered two different disease entities [1, 8]. Both may affect mucous membranes but can be distinguished by the morphology of the skin lesions. Contrary to EM, lesions in SJS/TEN consist of macules and atypical flat targets or detachment of large epithelial sheets of the skin affecting < 10% of the body surface area in SJS, 10–30% in overlap SJS-TEN and > 30% in TEN [1]. Drugs represent the main triggers of SJS/TEN, leading to an immune response driven by drug-antigen specific, clonally expanded cytotoxic CD8^+^ T cells [9]. Of interest however, Mp has not only been described as a trigger of EM, but also as a potential trigger [10–13] or co-trigger [14] of SJS/TEN.

Canavan et al*.* reviewed 202 documented cases of Mp-associated EM, SJS/TEN and mucositis, published between 1922 and 2013 [15]. Based on the observed clinical pattern, they proposed that mucocutaneous disease in the context of Mp infection constitutes a syndrome different from EM and SJS/TEN, and suggested the term *Mycoplasma pneumoniae*-induced rash and mucositis (MIRM) [15]. The concept of MIRM as a separate entity has since been adopted by different authors [16–21]. However, the concept has been rejected by others [3] and so far, there is no consensus on MIRM as a separate entity, nor has this concept been validated in further studies.

In contrast to HAEM, the pathophysiology of Mp-associated EM remains elusive. Here, we present the case of a patient with Mp infection and mucocutaneous disease characteristic of EMM. A characterization of lesional T cell responses in Mp-associated EMM has not been previously reported.

## Case description

**Fig. 1:**
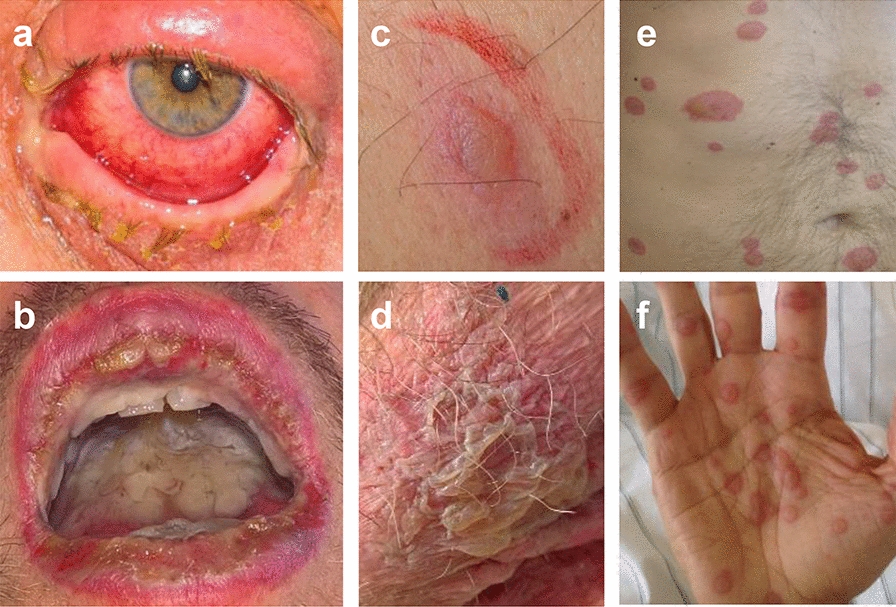
Involvement of different cutaneous and mucosal sites.** a** Conjunctivitis. **b** Erosive stomatitis and cheilitis. **c** Example of an early cutaneous blister. **d** Confluent area of epithelial detachment at the scrotal skin. **e**, **f** Widespread distribution of cutaneous lesions over the trunk (**e**) and extremities (**f**)

**Fig. 2:**
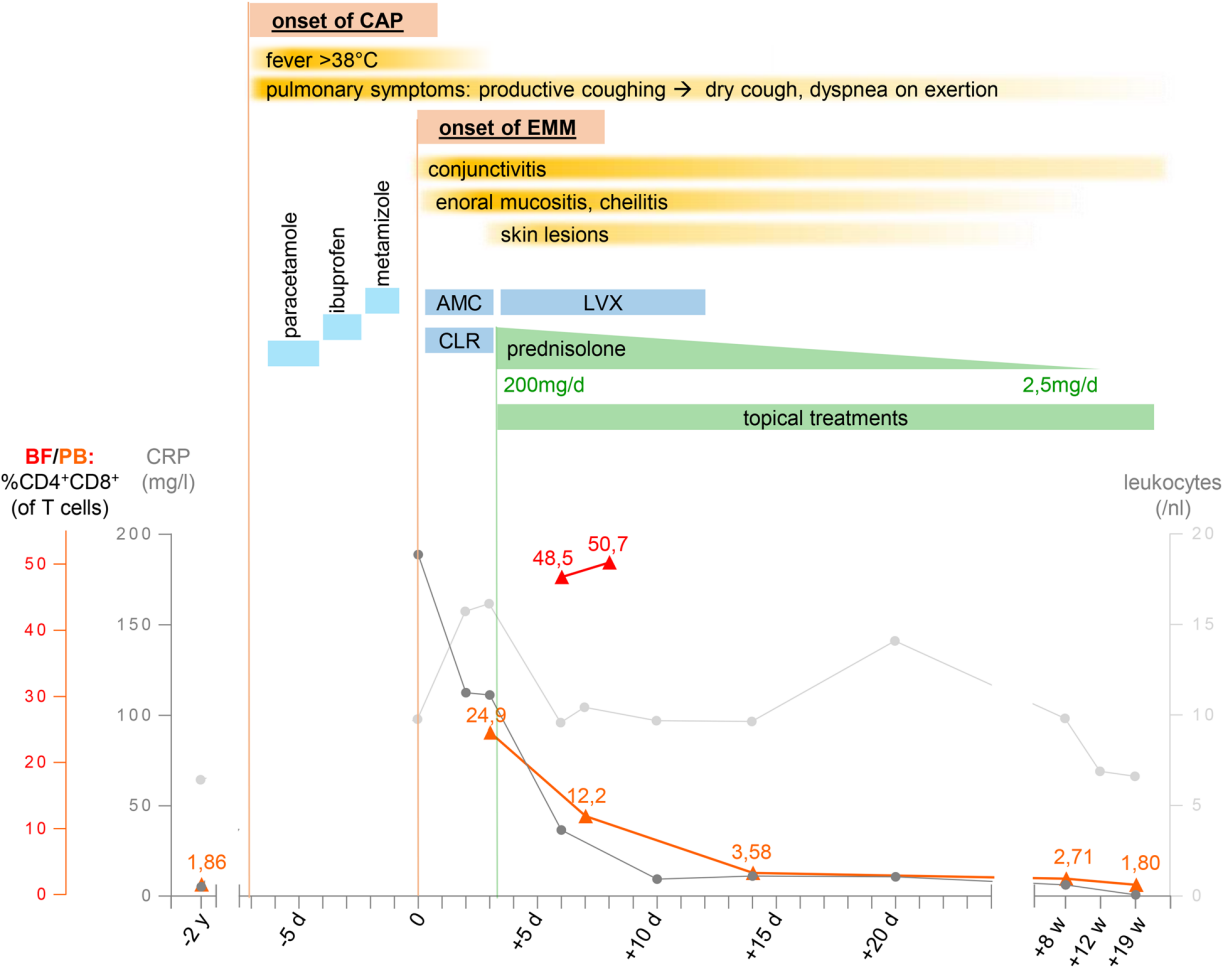
Timeline. Timeline of symptoms, drug exposure and treatment, including C-reactive protein (CRP) levels (dark grey line, normal range: < 5 mg/l, scale on the left side), total leukocyte count (light grey line, normal range: 3.9–10.5 /nl, scale on the right side) and percentage of CD4^+^CD8^+^ T cells (among total T cells) in peripheral blood (PB, orange) and blister fluid (BF, red). When percentage of CD4^+^CD8^+^ T cells was determined by two panels at the same day, the mean was calculated. Dosage of medication was 2 × 875/125 mg/d for amoxicillin-clavulanic acid (AMC), 2 × 250 mg/d for clarithromycin (CLR) and 2 × 500 mg/d for levofloxacin (LVX). Paracetamole, ibuprofen, and metamizole were taken successively, however, exact dosage could not be evaluated retrospectively. Further abbreviations: CAP: community-aquired pneumonia, EMM: Erythema multiforme majus, d: day, w: week, y: year

A 50-year-old man of European descent presented to the emergency department with a six-day history of productive cough with putrid secretion, fever up to 39 °C and a pounding headache. C-reactive protein (CRP) levels were elevated (188.7 mg/l, normal range < 5 mg/l), and chest X-Ray showed a slight infiltration in the left lower lobe. A diagnosis of non-severe community acquired pneumonia (CAP) was established. Oral treatment with amoxicillin/clavulanic acid and clarithromycin was prescribed and the patient was discharged. Two days later, he presented again to the emergency department. His condition had worsened, and he had developed severe erosive stomatitis, cheilitis and conjunctivitis with photophobia on both eyes (Figs. 1a, b, 2). According to the patient, conjunctivitis was observed prior to the first dose of oral antibiotics. He also complained of dysuria (urethritis) and rapidly developed vesiculobullous lesions on his trunk (first lesions), palms, and the scrotum (Fig. 1c–f). He was admitted to the infectious diseases ward. Antibiotic treatment was changed to levofloxacin, and due to the severity and rapid expansion of the mucocutaneous lesions, a supportive treatment with intravenous prednisolone was initiated by the consultant dermatologist (Fig. 2).

The medical history revealed that the patient had previously suffered from recurring respiratory tract infections, mainly bronchitis, up to five times per year. He had known allergies to grass-pollen and house dust mite with mild symptoms of allergic rhino-conjunctivitis. Of note, he had previously suffered from recurring enoral aphthous ulcers and recurring conjunctivitis in the past, the latter of which almost exclusively occurred in conjunction with respiratory infections. The family history revealed that his father, sister and son also suffered from recurring aphthous stomatitis. Immunological testing performed two years prior to the onset of mucocutaneous disease had not shown abnormal findings, with the exception of an isolated mannose-binding lectin deficiency (37.6 ng/ml; values > 50 ng/ml were considered normal) and slightly elevated serum levels of serum IgE (368.8 kU/l, values < 100 kU/l were considered normal).

In the days prior to presenting to the emergency department, the patient had taken the following medication; paracetamol (started six days prior to conjunctivitis, which was the first sign of mucocutaneous disease), ibuprofen (started four days prior to conjunctivits) and metamizole (started two days prior to conjunctivitis) (Fig. 2), a non-opioid analgesic commonly used in Germany but not available in all countries. He reported that he had taken paracetamol several times in the past without any adverse reactions to the drug. In contrast, he reported that it was his first-time exposure to ibuprofen and metamizole. Extensive microbiological and virological testing revealed weakly positive polymerase chain reaction (PCR) results for *Bordetella parapertussis (B. parapertussis)* in pharyngeal swabs, positive *Mycoplasma pneumoniae* serology and subsequent seroconversion (on admission: IgM 11.2, IgG negative; seven weeks later: IgM 35.0, IgG 19.1, values < 8.5 were considered normal) and marginally positive Human Herpesvirus 6 (HHV6)-IgM serology. Neither of these pathogens (*B. parapertussis*, Mp and HHV-6) could be detected by PCR in cutaneous blister fluid (BF). All other microbiological and virological analyses, including HSV-1/2 (PCR in peripheral blood (PB), BF, throat wash and eye smear negative, HSV1/2-IgM and IgG negative, serology negative also 2 years before), Epstein-Barr virus (EBV, DNA in PB 2260 copies/ml, limit of detection 1000 copies/ml, PCR in BF and throat wash negative, EBNA1-IgG 72,9; VCA-IgG > 750; EBV-IgM negative, tested twice 3 days apart), cytomegalovirus (CMV, PCR negative in PB, BF and throat wash), were not indicative of infection or reactivation.

The clinical presentation was characteristic of EMM, with mainly round target lesions showing central blistering and mucosal involvement of two mucosal sites (oral and ocular mucosa) (Fig. 1a, b). As there was no indication of recent HSV infection/reactivation and neither HHV-6, nor *B. parapertussis* have been reported as causes of EMM in the literature, Mp was considered the most likely trigger of mucocutaneous disease. Drugs have also been associated with EM [6], however, in retrospect these associations were often misclassified [22]. Therefore, drugs may not be considered likely triggers in a patient with EM lesions. In our patient, antibiotics could be excluded as causative triggers, since first symptoms (conjunctivitis) appeared prior to first exposure. Since the patient had been previously exposed to paracetamol without adverse reactions, this drug was also considered an unlikely trigger of the eruptions. Ibuprofen and metamizole, which were taken four days (ibuprofen) and two days (metamizole) before onset of conjunctivitis, cannot be completely ruled out as (co-) triggers—especially as it has been reported that Mp and non-opioid analgesics might also synergistically trigger disease [14]*.* Lymphocyte transformation testing (LTT) to assess for potential drug involvement was not conclusive when performed during the acute phase, as the positive control tested negative, potentially due to systemic high-dose corticosteroid (CS) treatment, and it did not retrieve positive results for any of the drugs four months after the acute phase. LTT often produces negative results after the acute phase and, therefore, it does not exclude drug causality [23].

The skin lesions as well as stomatitis and cheilitis slowly receded over the course of several weeks on symptomatic treatment and systemic CS. Pneumonic infiltration in chest X-ray had also largely dissolved at the time of discharge. In contrast, ocular lesions persisted and required prolonged treatment with topical CS and locally administered cyclosporine. The patient also reported a persistent dry cough over five months after discharge, as well as exertional dyspnea (which he had not experienced before) and pulmonary function test abnormalities (hyperinflation and airflow obstruction) that did not respond to treatment with systemic or inhaled CS and long-acting beta-2 agonists and were still present 1.5 years after the acute phase.

**Fig. 3:**
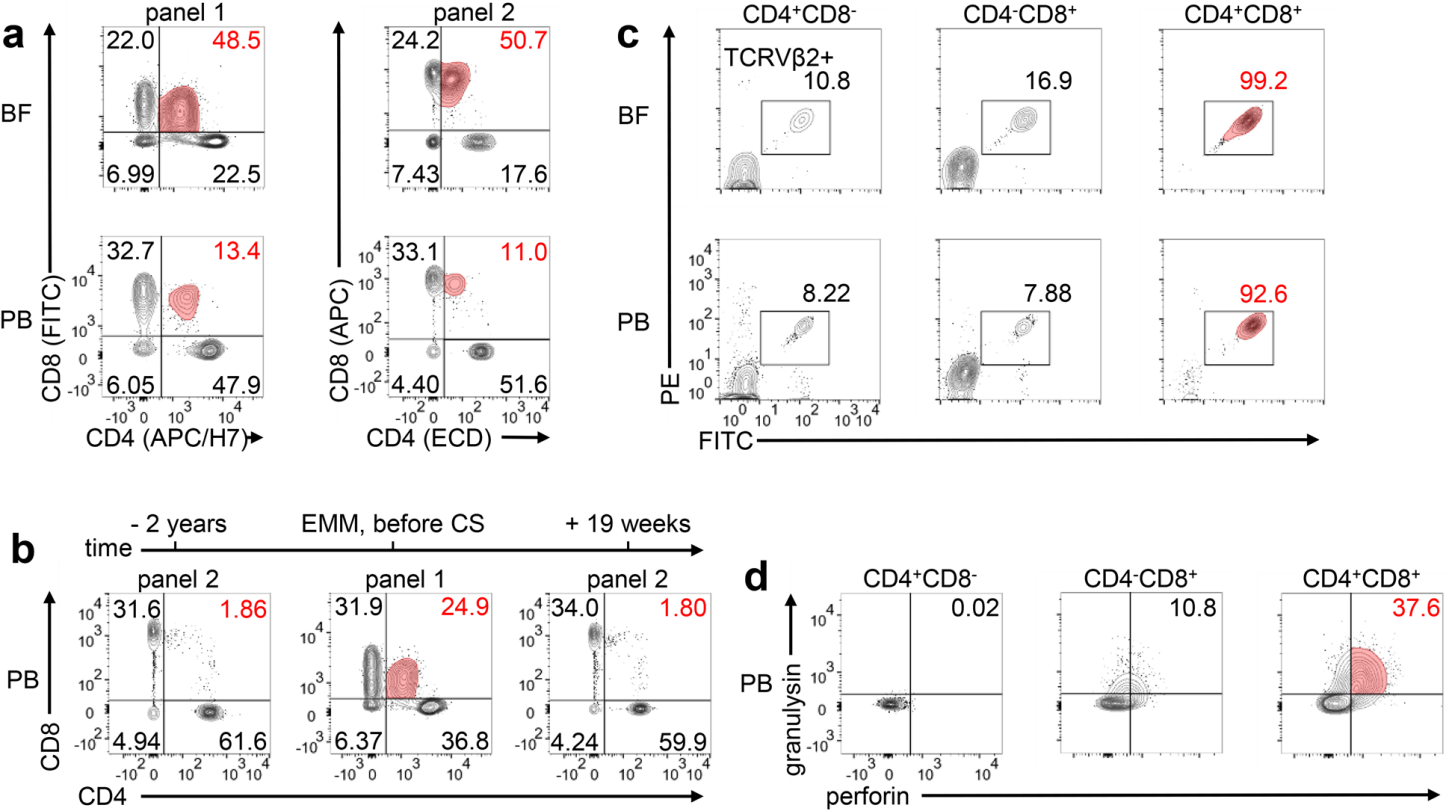
Detection of a clonally expanded CD4^+^CD8^+^ T cell population in blister fluid and peripheral blood. **a** CD4^+^CD8^+^ T cell frequencies within blister fluid (BF) and peripheral blood (PB) in two different flow cytometry staining panels (panel 1 and panel 2) 3–5 days after the initiation of corticosteroid (CS) treatment. **b** Frequencies of CD4^+^CD8^+^ T cells within PB 2 years prior to onset of disease, during the acute phase before initiation of CS treatment and 19 weeks after the acute stage. **c** Flow cytometry analysis of the frequency of TCRVβ2^+^ cells among CD4^+^CD8^−^, CD4^−^CD8 + and CD4^+^CD8^+^ T cell subsets at day 5 after initiation of CS treatment. Antibody against TCRVβ2 was labeled to FITC and PE at equal amounts. **d** Flow cytometry analysis of the frequency of PB T cells expressing the cytotoxic mediators granulysin and perforin among CD4^+^CD8^−^, CD4^−^CD8^+^ and CD4^+^CD8^+^ T cell subsets*,* assessed 10 days after initiation of CS treatment. The most relevant findings are highlighted in red

In order to better characterize the immunological changes, we analyzed the immune cell composition in PB and in cutaneous BF. Flow cytometry analyses on day five after initiation of CS treatment revealed that the inflammatory infiltrate in blisters was dominated by neutrophils (52%) and T cells (32%), with only minor representation of monocytes (6.9%), eosinophils (3.5%) and Natural Killer (NK) cells (1.5%). B cells (0.08%) were virtually absent in BF. We found that approximately 50% of BF T cells were double positive for CD4 and CD8 (48.5% three days, and 50.7% five days after initiation of CS treatment, Fig. 3a). A similarly expanded CD4^+^CD8^+^ T cell population was also detected in the patient’s PB (24.9% of all T cells before CS treatment, Fig. 3b; 13.4% (panel 1) or 11.0% (panel 2) five days after initiation of CS, Fig. 3a). This finding was verified by independent staining panels (Fig. 3a), largely excluding technical artefacts. CD4^+^CD8^+^ T cells belonged to the CD4^low^CD8^high^ subgroup of CD4^+^CD8^+^ T cells (Fig. 3a, b) and therefore likely might have derived from mature CD8^+^ T cells [24, 25]. TCRVβ clonotyping revealed that nearly all of the CD4^+^CD8^+^ T cells were TCRVβ2^+^ cells (99.2% in BF, 92.6% in PB, Fig. 3c), indicating a mono- (or oligo-) clonal expansion of the CD4^+^CD8^+^ T cells. A previous assessment two years before the onset of disease had shown a normal percentage of CD4^+^CD8^+^ T cells in PB (1.86% of T cells, Figs. 2, 3b). Over time, and potentially under the influence of systemic CS, which are known to decrease T cell activation and proliferation [26], the population size of CD4^+^CD8^+^ T cells in PB gradually declined to baseline levels (Figs. 2, 3b), along with the regression of mucocutaneous lesions (Fig. 2). We therefore hypothesize that this clonally expanded CD4^+^CD8^+^ T cell population was involved in disease pathophysiology in our patient.

Granulysin has been identified as an important effector molecule in bullous skin disorders mediated by cytotoxic T cells [27–29], including EMM [27, 28]. CD4^+^CD8^+^ T cells in BF in our patient expressed high levels of granulysin, along with perforin, and the frequency of cells expressing these cytotoxic markers among CD4^+^CD8^+^ was higher than among CD4^+^ or CD8^+^ single positive T cells (37,6% of cells among vs. 10,8% among CD4^−^CD8^+^ and 0,02% among CD4^+^CD8^−^ T cells, Fig. 3d), further indicating a pathogenic role of these cells in disease pathophysiology.

BF T cells displayed a highly activated (CD69^+^, HLA-DR^+^, CD11a^+^), highly differentiated (CD28^−^, CD57^+^) and Natural Killer T (NKT) cell -like (CD16/56^+^) phenotype (Table 1). Their counterpart population in PB displayed a similar phenotype, yet with different expression patterns of the activation marker CD69 and CD45RA (Table 1).

**Table 1:** Phenotype of T cells in blister fluid (BF) and peripheral blood (PB)

	Total CD3^+^		CD4^+^CD8^−^		CD4^−^CD8^+^		CD4 ^+^CD8^+^	
	BF	PB	BF	PB	BF	PB	BF	PB
TRM cell marker								
CD69^+^	68.4	3.30	67.4	0.52	66.9	5.91	68.2	1.26
CD69^+^CD103 ^+^	6.23	*NA*	3.56	*NA*	8.33	*NA*	6.91	*NA*
MAIT cell marker								
MR1^+^ (5-OP-RU)	1.25	2.09	0.50	0.28	3.16	4.36	0.34	0.46
NKT cell marker								
CD16/56^+^	64.6	24.4	1.41	1.14	67.2	36.3	87.9	86.1
Memory marker								
CD45RA^+^	11.5	68.2	0.67	62.7	20.7	78.4	10.6	61.1
Naive (CD45RA^+^CCR7 ^+^)	*NA*	41.3	*NA*	62.4	*NA*	32.8	*NA*	4.06
TEMRA (CD45RA^+^CCR7^−^	*NA*	26.9	*NA*	0.27	*NA*	45.6	*NA*	57.0
TCM (CD45RA^−^CCR7^+^)	*NA*	10.0	*NA*	20.7	*NA*	1.08	*NA*	0.55
TEM (CD45RA^−^CCR7^−^)	*NA*	21.8	*NA*	16.6	*NA*	20.6	*NA*	38.4
Activation/differentiation marker								
CD69^+^	68.4	3.30	67.4	0.52	66.9	5.91	68.2	1.26
HLA-DR^+^	37.3	19.0	25.4	4.76	24.6	20.3	52.6	65.7
CD11a^high^	81.5	42.7	46.0	13.2	84.3	61.3	94.7	95.6
CD57^+^	33.1	25.9	3.77	2.46	33.9	38.6	48.0	77.3
CD28^+^	32.1	69.2	99.2	99.0	34.1	53.8	0.70	5.33

Phenotypic flow cytometry analyses were performed 3–5 days after initiation of CS treatment. *NA *not assessed. Numbers represent proportions (%) of cells expressing the respective markers among total T cells (CD3^+^) or among a subset of T cells (CD4^+^CD8^−^ T cells, CD4^−^CD8 ^+^ T cells or CD4^+^CD8^+^ T cells)

More than half (57.0%) of CD4^+^CD8^+^ T cells in PB displayed a “T effector memory RA” (T_EMRA_, CCR7^−^CD45RA^+^) phenotype and were negative for CD69, whereas most BF CD4^+^CD8^+^ T cells did not express CD45RA and were CD69^+^ (Table 1). Only a minority of BF T cells was CD69^+^CD103^+^ (6,23% of total BF T cells, 6,91% of CD4^+^CD8^+^ BF T cells, Table 1), indicating that BF T cells did not represent “classical” long term Tissue Resident Memory T cells (T_RM_) of the epithelium [30], which have been previously implicated as potential triggers of tissue-specific restriction of symptoms in mucocutaneous diseases such as SJS/TEN [31]. Mucosal-Associated Invariant T (MAIT) cells, a semi-invariant T cell population that has been shown to display high cytotoxicity against bacterially infected epithelial cells [32] were also present only in low frequencies (1,25% of total BF T cells, Table 1).

## Conclusions

To the best of our knowledge, this is the first report of a large clonal expansion of CD4^+^CD8^+^ T cells in BF and PB of a patient with Mp-associated EMM. In the published literature, we could only find one other report describing BF immune cells in mucocutaneous disease in the context of Mp infection, which reported *“elevated CD4*^+^ */CD8*^+^ *(697/558* × *10*^*5*^/L*) T cells with absence of B cells”* in a pediatric patient with widespread epithelial detachment of the skin, reminiscent of SJS/TEN [33]. This report did not provide primary flow cytometry data and lacked further phenotypical characterization of T cells.

CD4^low^CD8^high^ T cells have been studied in the context of various viral infections such as HHV-6 [34], EBV [35, 36] and CMV [36] and there is solid published evidence that stimulation of CD8^+^ T cells via their TCR in combination with CD28 costimulation, but none of those signals alone, can lead to de novo expression of CD4 [37–40]. The role of other signals in this process and the stability of CD4 expression is unknown. If CD4/CD8 co-expression is of direct pathophysiologic relevance remains unclear. In line with our findings of higher cytotoxic mediator content in CD4^+^CD8^+^ cells (Fig. 3d), it has been found, that ligation of CD4 augments the cytotoxic potential of CD4^low^CD8^high^ T cells [39, 41]. Interestingly, CD4^+^CD8^+^ carbamazepine-specific T cell clones could be generated from patients with carbamazepine hypersensitivity [42]. Some of these clones—in contrast to CD4^+^ or CD8^+^ single positive clones—displayed drug antigen-specific proliferation even in the absence of antigen-presenting cells or the presence of MHC class I and II blocking antibodies in vitro [42].

Extrapulmonary Mp manifestation in general can be classified according to different pathomechanisms as of *i*) a direct type (bacterium present at the site of inflammation), *ii*) an indirect type (bacterium not present at the site of inflammation) and *iii*) a vascular occlusion type [43]. Direct culture of Mp from vesicular skin lesions has been reported in several early case descriptions of Mp-associated EM [44] and SJS/TEN [45, 46], pointing towards a direct bacterial involvement in the pathophysiology. However, Mp was not detectable via PCR (targeting the Mp P1 adhesion gene) in lesional biopsies of patients with Mp-associated EM in a more recent study [7] and indirect pathomechanisms such as polyclonal B-cell activation, cross-reacting autoantibodies resulting from molecular mimicry, akin to Mp-associated Guillain-Barré syndrome, immune complex deposition and complement activation, have all been discussed and seem to be favored in the current literature [15–17, 43, 47, 48]. However, there is no direct evidence for any of these pathomechanisms in the literature. Our observation that lesional T cells were clonally enriched for one TCRVβ family and expressed cytotoxic molecules like granulysin and perforin, indicates a clonal T cell response directed against a defined antigen, similar to what has been observed in HAEM and in drug-induced SJS/TEN. Furthermore, the majority of the CD4^+^CD8^+^ T cells showed a T_EMRA_ phenotype (CCR7^−^CD45RA^+^) in PB, but nearly all of the CD4^+^CD8^+^ T cells had lost CD45RA in BF, which has been reported for CD8^+^ T_EMRA_ upon antigenic encounter [49]. This finding supports the hypothesis that circulating CD4^+^CD8^+^ T_EMRA_ were recruited to mucosal and epithelial sites, where they downregulated CD45RA expression upon exposure to a defined antigen. This antigen could be an antigen of Mp, a neo- or autoantigen, or a viral or drug-derived antigen, in which case Mp would represent a co-stimulus rather than the primary cause of disease. Identifying the nature and the source of the causative antigen will be a critical step towards a targeted treatment.

No general conclusions can be drawn from observations in a single patient. However, in rare conditions such as Mp-associated EMM, observations made in single cases might be critical to generate hypotheses, disseminate knowledge and spur further systematic research.

## Material and methods

### Cell isolation and flow cytometry

Flow cytometry analyses of BF and PB (T) cells were performed in the diagnostic laboratory (Labor Berlin—Charité Vivantes GmbH) and in the research laboratory of our institution, according to standard protocols for isolation and surface staining of immune cells. BF immune cells were classified by granularity and size (side and forward scatter area) and expression levels of CD45, CD14 (monocytes), CD16/CD56 (neutrophils, proinflammatory monocytes, NK/NKT cells), CD19 (B cells), CD3 (T cells) following standard gating strategies used in routine diagnostics. T cells were then further characterized as shown in Table 1. TCRVβ clonotyping was performed using the IOTest Beta Mark TCR Vβ Repertoire Kit (Beckman Coulter). Fluorophore-conjugated 5-(2-oxopropylideneamino)-6-D-ribitylaminouracil (5-OP-RU)-loaded Major Histocompatibility Complex class I related molecule 1 (MR1) tetramers were used to identify MAIT cells, 6-formylpterin (6-FP)-loaded MR1 tetramers were used as a negative control. For analysis of granulysin and perforin expression (Fig. 3d) peripheral blood mononuclear cells (PBMC) were cultured in a humidified incubator in the presence of brefeldin A and monensin for 2 h before intracellular cytokine staining. Cells were not restimulated with Phorbol-12-myristat-13-acetat (PMA)/Ionomycin, to prevent PMA/Ionomycin induced downregulation of the CD4 molecule and secretion of granulysin and perforin. All flow cytometry analyses were performed on fresh PBMC processed immediately or kept at 4 °C overnight. Flow cytometry was performed on a BD FACS Canto II cytometer or Beckman Coulter 10-color Navios. Data was analyzed using FlowJo software Version 10 (Treestar).

## Data Availability

The datasets of this report are available from the corresponding author on reasonable request.

## Abbreviations

BF: Blister fluid
*B. parapertussis*: *Bordetella parapertussis*
CAP: Community-aquired pneumonia
CMV: Cytomegalovirus
CRP: C-reactive protein
CS: Corticosteroid(s)
EBNA1: Epstein–Barr nuclear antigen 1
EBV: Epstein-Barr virus
EM: Erythema multiforme
EMm: Erythema multiforme minus
EMM: Erythema multiforme majus
HHV-6: Human Herpesvirus 6
HAEM: HSV-associated erythema multiforme
HLA: Human Leukocyte Antigen
HSV: Herpes Simplex virus
LTT: Lymphocyte transformation testing
MAIT cell: Mucosal-Associated Invariant T cell
MIRM: *Mycoplasma pneumoniae*-induced rash and mucositis
Mp: *Mycoplasma pneumonia*
MR1: Major Histocompatibility Complex class I related molecule 1
NK cell: Natural Killer cell
NKT cell: Natural Killer T cell
PB: Peripheral blood
PBMC: Peripheral blood mononuclear cells
PCR: Polymerase chain reaction
PMA: Phorbol-12-myristat-13-acetat
SJS: Stevens-Johnson syndrome
SJS/TEN: Stevens-Johnson syndrome/Toxical Epidermal Necrolysis
TCR: T cell receptor
TEN: Toxical Epidermal Necrolysis
T_EMRA_: T effector memory RA
T_RM_: Tissue Resident Memory T cells
VCA: Viral-capsid antigen
